# Optimizing T Cell Expansion in a Hollow-Fiber Bioreactor

**DOI:** 10.1007/s40778-018-0116-x

**Published:** 2018-02-27

**Authors:** Brian Nankervis, Mark Jones, Boah Vang, R. Brent Rice, Claire Coeshott, Jim Beltzer

**Affiliations:** Terumo BCT, Inc., 11158 West Collins Avenue, Lakewood, CO 80215 USA

**Keywords:** Bioprocessing, Cell therapies, Hollow-fiber bioreactor, Scale up, T cell

## Abstract

**Purpose of Review:**

Recent developments in regenerative medicine have precipitated the need to expand gene-modified human T cells to numbers that exceed the capacity of well-plate-based, and flask-based processes. This review discusses the changes in process development that are needed to meet the cell expansion requirements by utilizing *hollow-fiber bioreactors*. Maintenance of cell proliferation over long periods can become limited by unfilled demands for nutrients and oxygen and by the accumulation of waste products in the local environment.

**Recent Findings:**

Perfusion feeding, improved gas exchange, and the efficient removal of lactate can increase the yield of T cells from an average of 10.8E +09 to more than 28E +09 in only 10 days.

**Summary:**

Aggressively feeding cells and actively keeping cells in the bioreactor improves gas exchange and metabolite management over semi-static methods. The ability to remove the environmental constraints that can limit cell expansion by using a two-chamber hollow-fiber bioreactor will be discussed.

## Introduction

The growing field of cell therapy requires increasingly complex and varied processing, expansion, and manipulation of cell products to support the array of intended therapeutic targets. Mesenchymal stromal cells (MSCs) derived from bone marrow aspirates may be expanded to support the immunosuppression needs of graft-versus-host disease patients [1]. Monocyte populations derived from a leukapheresis product can be driven through a maturation process that produces dendritic cells which can be used as potential therapeutic cancer vaccines [2]. Perhaps the most notable recent advancement is the expansion of chimeric antigen receptor T cells (CAR T cells) targeted at specific hematological cancers [3•]. Though the details of each of these example differ, common considerations arise with respect to the environment required by the specific cell population. What is the relative importance of media component concentrations, waste removal, and autocrine/paracrine effects? How do we provide sufficient oxygen and pH regulation? How important or detrimental is cell-to-cell contact? This paper briefly explores such questions as they relate to the needs of a rapidly expanding T cell population in the Quantum® Cell Expansion System (from Terumo BCT). Process improvements have supported lower seeding densities, more sustained log-phase growth, and higher yields.

Hollow-fiber bioreactors were originally designed for kidney dialysis and have been used for over 20 years to produce monoclonal antibodies and other protein biologics [4]. A hollow-fiber bioreactor separates the culture space into two compartments using a semi-permiable membrane. Cells are maintained on one side of the membrane. Metabolites, gas, and nutrients continuously diffuse through the membrane, providing the cell population with the desired culture conditions. Small diffusion distances (200 μm diameter for each hollow fiber) [5] provide greater efficiency of gas and nutrient transfer than traditional culture flasks. The fluid compartmentalization characteristic of hollow-fiber bioreactors may be seen as an advantage over stirring and wave motion bioreactors due to decreased physical stress on the cells [6].

Culture conditions in the hollow-fiber bioreactor need to be optimized to match as closely as possible the physical, chemical, and biological environment that is optimum for each cell type [7]. Temperature, nutrients, gas, waste products, and metabolite concentrations are just a few of the parameters to be considered. Hollow-fiber bioreactors can be scaled up and can be used to culture any cell type, but are generally more suited to adherent cells.

Hollow-fiber bioreactors are commercially available from many sources including FiberCell Systems, Cellab, and Terumo BCT. The Fibercell Hollow-Fiber Perfusion Bioreactor System is a single-use high-density culture system consisting of a cartridge and a single-use flow path and pump module which are placed in an incubator. A wide variety of membranes are available from FiberCell Systems. The different membranes allow different flow rates and molecular weight cutoffs making the system suitable for numerous applications including monoclonal antibody production and the continuous processing of primary stem cells [8]. The Cellab® Bioreactor System is a lab-scale and small-scale semi-automated, functionally closed system that can be used for process optimization by testing up to five different culture conditions. The system consists of a docking station, hollow-fiber bioreactor with integrated gas transfer modules, and automated process control. The bioreactor can be scaled up to a single bioreactor with 2500 cm^2^ of surface area [9].

## Introduction to the Quantum

**Fig. 1:**
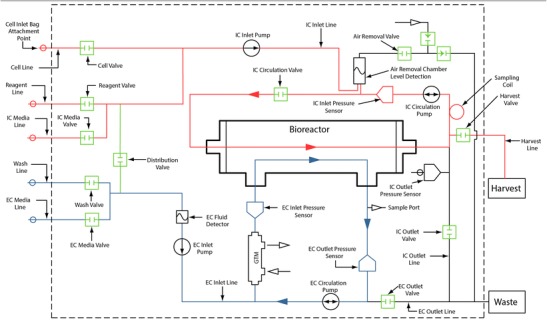
The Quantum System hydraulic layout with the IC loop shown in *red* and the EC loop shown in *blue.* (Copyright, Terumo BCT, Inc. 2017. Used with Permission)

The Quantum® Cell Expansion System (Quantum) is a commercial, functionally closed single-use hollow-fiber bioreactor device. The dual-loop fluidics design has an intra capillary (IC) loop and an extra capillary (EC) loop separated by a semi-permeable membrane. Cells cultured in the IC loop are continuously perfused via the EC loop, which provides access to fresh medium while simultaneously removing permeable waste products. Gas exchange is mediated by the circulation of the EC loop fluid through a gas transfer module and back through the bioreactor [10••] (Fig. 1 Quantum hydraulic layout).

The Quantum has been shown to be effective for culturing adherent cells including bone-marrow-derived mesenchymal stromal cells (MSCs) [11, 12••, 13•] and adipose tissue derived MSCs [14], which may be destined to be used in cell therapy applications. MSCs produced in the Quantum have been shown to be of a comparable quality to flask-grown cells [15•].

The dramatic therapeutic advances in immuneotherapy have elevated the need to culture primary human T lymphocytes, which are nonadherent in culture. The clinical manufacture of CAR T cells is an extremely complex process whereby the patient's own T cells are modified to express the CAR, usually via viral transduction, and then expanded before being reintroduced to the patient. The CAR enables the T cells to recognize a specific antigen present on cancer cells [3•]. To grow sufficient numbers of T cells for clinical trials, peripheral blood mononuclear cells can be stimulated to proliferate in static culture in flasks by co-culture with beads coated with anti-CD3 and anti-CD28 monoclonal antibodies in a 3:1 bead-to-cell ratio. In one study, the median fold expansion of cells was 84-fold in 14 days. Using the G-Rex gas-permeable flasks with improved gas permeability in a semi-closed system allows the cells to grow to a higher density than traditional static culture with 3-fold to 4-fold reduction in the amount of medium required [16].

Here, we examine process development for the expansion of CAR T cells in the Quantum. With clinical dosing of CAR T cells potentially requiring multiple doses of 1E +09 cells [17], our goal was to maximize the output of the T cell expansion. The first-generation protocol was based on static culture conditions such as found in a well-plate-based, flask-based, or G-Rex system. The second-generation protocol more completely utilized the perfusion capabilities of the Quantum. The effects of these environmental changes in the culture conditions between the first- and second-generation protocols will be discussed.

### First-Generation T Cell Expansion Approaches in Hollow-Fiber Bioreactors

The first-generation T cell expansions in the Quantum cell expansion system have been described in detail [10••]. Briefly, one hundred million lymphocytes from a mononuclear leukapheresis product (leukopak) collected from healthy human donors using the Spectra Optia Apheresis System were used as the starting material for the expansion. The apheresis product contains a variety of mononuclear cells, including predominantly monocytes and lymphocytes, with some remaining platelets, plasma, and red blood cells (RBCs). Activation of T lymphocytes was achieved using anti-CD3 and anti-CD28 monoclonal antibodies. This T cell activation is required for T cell proliferation, cytokine production, and expression of the interleukin-2 (IL-2) receptor. CD3/CD28-coated Dynabeads® called “Human T-Activator” represent a convenient method for T cell activation. Therefore, the cells were activated with CD3/CD28 Dynabeads using a 1:2 bead-to-cell ratio in 100 mL of medium containing interleukin (IL)-2 and IL-7. Upon preparation of this suspension, the cells were immediately loaded into the Quantum and distributed throughout the IC loop of the bioreactor, where they were fed at a small, continuous rate (0.1 mL/min) with complete medium containing IL-2 (200 IU/mL) and IL-7 (5 ng/mL). Concurrent with feeding via the IC inlet pump, fluid in the EC loop was circulated through the gas transfer module at 100 mL/min by the EC circulation pump to support gas exchange. Simultaneously, the IC loop was circulated slowly and continuously using the IC circulation pump at 1 mL/min to ensure that cells throughout the IC loop also had access to the oxygenated medium (refer to Fig. 1 Quantum hydraulic layout to see the inlet and circulation pumps on both the IC loop and the EC loop). Additional feeding was provided on days 3, 6, and 9 by adding a bolus of 150 mL of complete medium containing both IL-2 and IL-7, during which the cells were also redistributed throughout the loop using a high IC circulation rate of 100 mL and bioreactor motion. Finally, starting at day 6, the bioreactor was rotated 180° each day to allow a subtle redistribution of the cells with the intention of preventing necrotic centers with increasing cell mass.

**Fig. 2:**
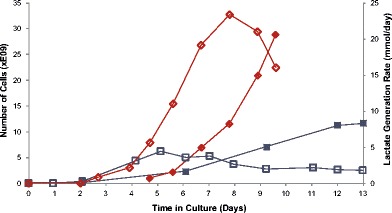
T cell expansion in Quantum. *Blue lines* represent run 2 of the first-generation protocol (2-1st Gen). *Solid blue boxes* indicate number of cells (in billions) and *open blue boxes* represent lactate generation rates (mM/day). *Red lines* represent run 3 of the second-generation protocol (3-2nd Gen). *Solid red boxes* indicate number of cells (in billions) and *open red boxes* indicate lactate generation rates (mM/day)

Several observations from the data obtained with the first-generation approaches provide insight into potential process improvements. After a 13-day expansion under this protocol, yields ranged from 7 to 14 billion cells with CD3+ T cells comprising greater than 90% of the cells, and viabilities ranging from 84% to 91% (see Table 1, runs 1–3 1st Gen for results from the first-generation runs using this protocol). Figure 2 shows representative cell proliferation data and lactate generation rates for both the first-generation protocol and the second-generation protocol. While the first-generation protocol shows cell numbers increasing after day 6, the lactate generation rate is decreasing during this time. In fact, the average doubling time for cells cultured on days 0–6 increases from 31 to 53 hours on days 6–13 when cultured using the original protocol (see Table 1). The slower growth rate may also reflect the relatively low feed rate of the original protocol (Table 1; and discussed further below). Additionally, the continuous feed stream (IC inlet) and IC circulation of the 1st Gen protocol allowed cells to collect in the open space at the end of the hollow-fiber bioreactor, which could allow cell death within the cell mass.

**Table 1:** Characterization of the first-generation protocol and second-generation protocol

Run	Fold expansion*	Total media feed (L)	Doubling time days 0–6	Doubling time day 6–harvest	Max cell density (cells/mL)	% CD3+ cells harvested	Viability (%)
1-1st Gen	450	2.4	33.9	36.7	72E + 06	98.5	91
2-1st Gen	280	2.4	27.9	72.5	65E + 06	98.8	91
3-1st Gen	117	2.2	30.8	50.1	43E + 06	90.9	84
1-2nd Gen	557	10.4	54.6	18.5	210E + 06	98.8	91
2-2nd Gen	478	15.3	34.9	20.1	245E + 06	99.7	92
3-2nd Gen	439	13.9	28.2	26.0	233E + 06	99.5	81

1-1st Gen to 3-1st Gen are the first-generation expansion protocol runs 1–3 and 1-2nd Gen to 3-2nd Gen are from the second-generation protocol runs 1–3. Fold expansion is the fold increase of CD3+ cells from beginning to end of the protocol. Total media feed is the amount of media supplied to each run in liters. Doubling time from days 0 to 6 of each run. Doubling time values day 6 through harvest for each run. Maximum cell density in the bioreactor was measured at the time of harvest. Yield of CD3+ cells at harvest (*expressed as a percentage*). Viability of each run at the time of harvest (*expressed as a percentage*)

The fold expansion appears to be directly correlated with the ratio of beads to CD3+ T cells, as could be expected. The first-generation protocol bead counts were dictated by total lymphocyte counts provided by a complete blood count (CBC), rather than CD3+ cell counts. Though an approach based on total lymphocyte counts may be more practical, the inadvertent limitation of lower bead-to-CD3+ T cell ratios suggests that proactively higher bead utilization could encourage greater expansion.

Finally, the total amount of medium used during the first-generation protocol expansion was approximately 2 L. This minimal feeding approach left the lactate levels above 16 mM and the pH at approximately 6.7 for the final third of the expansion. High concentrations of lactate may result in growth inhibition, decreased survival, and decreased cytokine secretion in primary cells [18]. While work in other bioreactors has clearly demonstrated the value of perfusion feeding for removal of the inhibitory metabolites lactate and ammonia [18], the perfusion capabilities of the Quantum system were largely ignored in this early protocol. However, with expansion yields from 90- to 500-fold, the first-generation protocol was equal to or better than early flask-based expansions [10••]. The first-generation protocol could be thought of as a proof of concept experiment.

### Second-Generation T Cell Expansion Approaches in Hollow Fiber Bioreactors

The second-generation of the Quantum system T cell protocol aimed not only for improvements stemming from the observations above, but also to provide greater robustness to the protocol. As a process simplification, the IL-7 was removed, though it had provided some improvement over IL-2 alone in the original protocol. Additionally, starting cell populations were purified and cryopreserved prior to use. Starting materials were derived from apheresis products; however, peripheral blood mononuclear cells (PBMCs) were further isolated from RBCs and platelets using elutriation (Elutra® Cell Separation System, Terumo BCT) and then cryopreserved. Elutriation is a process that separates particles based on their size, shape, and density. Elutriation is usually accomplished by a stream of liquid pumped in a direction opposite the direction of sedimentation.

Beginning with a cryopreserved PBMC fraction, beads were added in a 1:1 ratio with PBMCs and suspended in media. Determining the number of beads to add based on PBMCs rather than lymphocytes ensured that the beads-to-CD3+ T cell ratio was consistently higher than that used in the first-generation protocol.

The cells, while always inside the IC loop, also need to remain within the bioreactor (Fig. 1) whereas the fluid in the IC loop circulates. To keep the cells within the hollow fibers and not allow cells to leave the bioreactor, the circulation rate within the IC loop was decreased during both seeding and feeding. As the cells were seeded into the bioreactor, they were actively directed with the flow into the hollow fibers, rather than leaving them distributed throughout the loop. Additionally, medium fed into the IC loop was distributed into both ends of the bioreactor, which retained the cells in the fibers of the bioreactor. This was accomplished by directing the IC_circ_ pump in the opposite direction of the IC_inlet_ pump. Without these opposing pump directions, even a small IC inlet rate can result in cells leaving the bioreactor into header space where a growing cell mass can accumulate. Such a large mass of necrotic cells was observed in the outlet header in the first-generation protocol. By holding the cells within the bioreactor using opposing pump directions, cells were maintained in the bioreactor and not in the header space.

Avoiding cell collection within the header has the obvious advantage of limiting the formation of necrotic centers in this space. An additional advantage is that the cells are maintained where access to the gas supply is most efficient. It has been demonstrated elsewhere that O_2_ requirements increase dramatically as cell density increases [16]. By maintaining the cell population within the fibers, they have ready access through the semi-permeable hollow-fibers to the medium within the EC loop. In turn, the medium within the EC loop is provided continual access to the gas supply via circulation through the high surface area of the gas transfer module. To support the increased respiratory demands of larger cell populations, the circulation within the EC loop—and, therefore, through the gas supply—was increased progressively throughout the expansions described here, reaching a maximum of 300 mL/min.

While the first-generation expansion protocol included a small continuous addition of medium to the IC loop with several larger additions in the latter half of the expansion, the second-generation expansion protocol sought to more aggressively provide nutrient supply and waste removal. In fact, the amount of media used in the second-generation protocol was almost 5-fold more than the first-generation protocol  (see Table 1). Increasing the nutrients supplied to the rapidly expanding T cells was handled not just by the IC_inlet_ but also by the EC_inlet_. In the Quantum perfusion system, the IC_inlet_ is the preferred delivery route for larger proteins such as IL-2. When the rapidly dividing cells require more glucose than can be provided by increasing the IC_inlet_ rate, increasing the delivery of glucose to the cells may be accomplished simply by increasing the EC_inlet_ rate.

Growth factors and cytokines required for T cell expansion and thereby required to remain at a predetermined concentration with the cells in the IC loop could be washed out or dramatically diluted if they pass through the semi-permeable membrane of the hollow-fibers and into the EC loop. We tested the fate of IL-2 in our second-generation protocol. For practical purposes, the molecular weight cutoff for the hollow-fibers in the Quantum system is approximately 17 kDa. Although the molecular weight of IL-2 is 15,000 Da, it was retained in the IC loop and was minimally detected in the EC loop during the expansion (data not shown).

Another potential concern is cell packing. High cell concentrations within the IC loop can cause the cells to pack together and could result in the formation of necrotic areas within the hollow-fibers. The second-generation protocol increased the frequency of the cell redistribution and feed to once a day for the first 4 days and then twice daily until the end of the expansion. Aggressively pushing those cells back into the hollow-fibers, or increasing the reverse pumping of the IC_circ_ pump, could become detrimental. For those reasons, an IC_inlet_ feed rate of greater than 0.4 mL/min is not recommended.

When the feeding requirements of the culture exceed 0.4 mL/min, feeding can be accomplished by the EC loop where metabolites cross the semi-permeable membrane of the hollow-fiber. The EC_inlet_ feed rate may vary from 0 to 10 mL/min and the EC_circ_ rate may be increased up to 300 mL/min. Increasing the EC_inlet_ rate effectively increases the supply of glucose and the removal of lactate from the IC loop. Increasing the EC_circ_ rate homogenizes the culture environment and increases the supply of O_2_. Feeding via the EC_inlet_ also allows for the use of a base medium which does not contain expensive cytokines and growth factors.

Cell mixing and redistribution is dramatically increased in the second-generation protocol. With obvious benefits for cell feeding and metabolite management, it also optimizes cell contact with the CD3/CD28 Dynabeads. T cells activated by a cognate antigen have been shown to undergo at least seven population doublings [16] after activation in static culture. Increasing the bead concentration and mixing of the cells improves the activation and the reproducibility of T cell expansions in the Quantum. Mixing by redistribution of the cells and by rotating the bioreactor are two methods which can be used to alter the macro-environment of the cells in the bioreactor.

Figure 2 provides a representative example of the improvements provided by the second-generation protocol. By providing better activation, better management of the cell population, better gas provision, better nutrient supply, and better waste removal, expansion was greatly improved in the early phases of the expansion (see Table 1). The decrease in lactate production, a measure of cellular metabolism, seen on days 7–10 of the second-generation expansion reflects room for further improvements. Regardless, yields have routinely doubled relative to that in the first-generation, while maintaining high viabilities of > 81% and improved CD3+ T cell purity of > 98.8%.

Using these improved feeding protocols can result in high cell densities within the hollow-fiber bioreactor. Cell densities of greater than 2E +08 cells/mL were achieved, an approximately 4-fold improvement over the first-genration protocol as shown in Table 1. Additionally, due to the unique configuration of the Quantum system, harvested cells are collected in a manageable volume of 700 mL indicating the efficiency of post-processing provided by this system.

## Conclusions

The Quantum® Cell Expansion System represents an automated solution for the culture and expansion of high numbers of human CAR T cells that may be utilized in the treatment of numerous types of cancer. The Quantum system is a hollow-fiber bioreactor with the ability to control the flow of liquids and metabolites in a two-chamber system separated by a semi-permeable membrane. The first-generation expansion protocol which was based on micro-well-plate culture conditions yielded relatively pure cultures of CD3+ T cells in the range of 7–14 billion cells. However, upon closer inspection, the culture is lagging as demonstrated by the declining lactate production and increasing doubling time after day 6 (see Table 1). Better activation, gas provision, nutrient supply, waste removal, and management of the cell population in the second-generation expansion protocol consistently resulted in improved cell growth. As shown in Table 1, the improvements of the second-generation protocol supported final resident cell densities in the 189 mL IC loop of over 2E +08  cells/mL, corresponding to yields that routinely doubled relative to those in the first-generation protocol. Upon harvest, cell densities in the final 700 mL product remained at a high density of 4E +07   cells/mL. All products maintained high viabilities of > 81% and improved CD3+ T cell purity of > 98.8% (Table 1). Further process improvements using the Quantum system are being explored.
